# A longitudinal analysis of generational shifts in professional men’s tennis across the open era

**DOI:** 10.1038/s41598-026-57291-x

**Published:** 2026-06-18

**Authors:** Firas Bayram, Annalisa Barla

**Affiliations:** 1 SnT, University of Luxembourg, Luxembourg, Luxembourg; 2 DIBRIS, University of Genova, Genoa, Italy; 3 MaLGa, University of Genova, Genoa, Italy

**Keywords:** Tennis evolution, Concept drift, Tennis dynamics, Sports analytics, Evolution, Mathematics and computing

## Abstract

Professional men’s tennis has transformed significantly since the Open Era began in 1968, with shifts in playing styles, competition levels, and match patterns driven by advances in technology, training, and rules. However, systematic quantification of these changes remains unexplored. This study examines approximately 198,000 ATP matches from 1968 to 2025 through network analysis and machine learning techniques, applying concept drift methods to characterize temporal changes and understand how the sport has evolved across eras and court surfaces. Key findings include greater overall competitiveness, with more frequent upsets and fewer one-sided wins, as top-player dominance has spread more evenly across the field. Matches have grown longer by about 16.5 minutes on average since 1991, serves have become more effective (with 1.7 more aces per match and 2.4% higher win rates on serve points), and return effectiveness has declined by 2.4%. Court surfaces show increasing similarity, with grass courts converging toward the pace of other surfaces. These findings quantify the sport’s shift toward a more powerful, endurance-based game, amid advancements in racket technology and player conditioning. This research demonstrates the power of machine learning to uncover the evolutionary patterns of the tennis game.

## Introduction

Tennis, celebrated for its blend of athleticism, strategy, and psychological resilience, has evolved dramatically since the advent of the Open Era in 1968. This period marked the integration of amateur and professional players, leading to increased global participation and commercialization. Technological advancements, such as the switch from wooden to composite rackets, have enabled greater power and spin^1^, while changes in court surfaces, from fast grass to slower hard and clay variants, have influenced the tempos of matches. These evolutions reflect broader trends in sports science, including improved conditioning programs that emphasize endurance and injury resistance^2^. As a result, the sport has moved away from short and aggressive points to longer rallies and multifaceted strategies, raising questions about how the modern game differs from previous eras^3^.

Physical and tactical demands on players have intensified markedly in recent decades. Tennis in the early Open Era emphasized serve-and-volley tactics, rewarding explosive speed, quick reflexes, and net play proficiency, while modern tennis prioritizes baseline consistency, exceptional court coverage, and sustained physical and mental endurance^4^. Studies have shown increases in rally lengths and cumulative match load, correlating with higher injury rates and extended recovery needs^5^. For instance, biomechanical analyses highlight how advanced string technologies allow for sharper angles and heavier topspin, altering stroke mechanics and forcing adaptations in player training^6^. These shifts redefine success metrics and also impact spectator engagement, with debates on whether slower games enhance or diminish the appeal of the sport^7^.

At the match level, tennis matches are structured in best-of-three or best-of-five sets, with outcomes mainly shaped by players’ technical skills, physical conditioning, and strategic decisions. The core elements of play—serve effectiveness, rally length, and point construction—vary considerably by surface, with hard courts producing fast-paced exchanges and clay courts favouring prolonged baseline rallies^8^. Although the scoring rules have remained largely unchanged since the Open Era, match dynamics have evolved substantially due to advances in racket technology, training methodology, and the progressive homogenization of court surfaces, producing measurable shifts in serve speed, rally duration, and recovery patterns.

Fortunately, tennis provides an ideal domain for quantifying these evolutionary trends due to its data-rich nature. Modern match-level datasets typically include serve and return statistics, point-by-point outcomes, player movement and shot selection data, as well as contextual information such as surface type, tournament level, and match round^9^. Comprehensive statistics have been systematically captured since the Open Era’s inception, covering metrics such as serve percentages, aces, break-point conversions, and match duration, offering granular insights into performance^10^. The digital era has amplified this through databases like the ATP Tour records, enabling retrospective analyses of thousands of matches across multiple decades and generational cohorts^11^. Such resources allow researchers to move beyond subjective commentary to evidence-based evaluations, identifying patterns across players, tournaments, and playing conditions^9^. This quantitative foundation is crucial for understanding how external factors, such as rule tweaks or equipment regulations, propagate through the sport^12^.

Despite these data opportunities, quantitative research capturing the holistic evolution of tennis remains limited. While expert commentary frequently highlights perceived changes such as increased baseline power, faster serves, extended rallies, these observations often lack rigorous quantitative validation. Existing works often isolate specific elements, such as match outcome prediction models^13^, serve velocity determinants^14^, or injury prevalence^5^, without examining how multiple performance dimensions interact and evolve simultaneously. For example, while some investigations compare eras through basic statistics, they rarely account for interconnected changes in competitiveness, surface effects, or strategic adaptations^15^. This piecemeal approach leaves a critical gap: a holistic, longitudinal framework to map the sport’s trajectory and quantify its multifaceted shifts over time.

Artificial intelligence (AI) techniques, particularly machine learning (ML), complemented by network analysis for a holistic view of player interactions, offer effective frameworks for analyzing temporal patterns in complex datasets^16^. Concept drift—a well-established paradigm in ML—describes temporal changes in the statistical properties of a data-generating process^17^. In practice, drift analysis distinguishes two complementary components: distributional shift, which captures changes in recorded match characteristics such as serve speed or match duration distributions (*P*(*X*))^18^, and concept drift, which reflects how the influence of these features on winning outcomes changes over time (*P(Y ∣ X)*)—for example, whether first-serve accuracy or surface type becomes more or less decisive^19,20^. Both components are routinely assessed through distributional comparison metrics, conditional probability estimation, and cross-period model evaluation. Applied to tennis, this analytical framework enables systematic quantification of how evolving contexts —such as technological innovations, training methodologies, and regulatory changes—reshape match statistics and their relationship to competitive outcomes across generations.

In this study, we conducted a comprehensive longitudinal analysis of nearly 198,000 ATP matches from 1968 to 2025, integrating network analysis, statistical modeling, and concept drift analysis. We examine shifts in competitive balance, performance metrics, and surface-specific characteristics, revealing increased parity, extended match times, and surface homogenization—a convergence in playing conditions across court types that, paradoxically, demands greater tactical versatility from individual players. This work contributes both a novel methodological framework for analyzing sports evolution and empirical insights into how professional tennis has changed over the decades, deepening our understanding of the forces shaping the modern game.

## Methods

To study the evolution of tennis over the past decades, we developed a comprehensive data-driven framework combining match-level statistics, competitive balance evaluation, and concept drift analysis. Our approach integrates temporal, feature-level, and surface-specific perspectives, enabling systematic investigation across multiple dimensions and granular levels.

### Tennis match data collection

Match data were obtained from the publicly available and widely used *Tennis Abstract* repository^21,22^, a compilation of official ATP tour-level match records, covering all tour-level men’s singles matches (Grand Slams, ATP Masters 1000, ATP 500, ATP 250) from 1968 to 2025. Several preprocessing steps were applied to ensure data quality. Date fields were validated against the expected format and score strings were parsed and validated against the standard set format (e.g., 6–3, 7–6(7)) using regular expressions. Domain constraints were enforced to flag logical inconsistencies—for instance, negative ace or double-fault counts, break points saved exceeding break points faced, or first-serve-in counts exceeding total service points. Additionally, a random sample of match records was manually cross-checked against the official ATP Tour website (https://www.atptour.com) to verify the accuracy of the data. Matches ending in retirement, walkover, or default—identified by “RET”, “W/O”, or “DEF” markers in the score string—were excluded from the competitive balance analysis, as these outcomes do not reflect the full competitive encounter. However, such matches were retained in network analyses, since the match outcome itself remains valid. For distributional shift and machine learning analyses, which require complete in-match statistics (aces, service points, match duration), matches with missing values in the relevant columns were excluded via listwise deletion; this implicitly removes most retirements and walkovers, as these typically lack recorded in-match statistics. After preprocessing, the final dataset comprised approximately 198,000 matches for competitive balance and network analysis (1968–2025) and approximately 98,000 matches with complete in-match statistics for distributional shifts and concept drift analysis (1991–2025).

Our analysis employs temporal windowing strategies determined by data availability and analytical objectives. Competitive balance metrics (upsets, heavy defeats, straight-set proportions) rely on match outcomes and player rankings, which have been systematically recorded since the Open Era began in 1968; we use decade-level windows across the full 1968–2025 period (*∼*198,000 matches), as decade-level grouping is standard in longitudinal tennis research—Radicchi ^23^, for instance, employed decade-based windows when studying historical tennis generations—and provides sufficient match volume per era (averaging *∼*33,000 matches) to capture sustained competitive patterns representative of distinct generations. In contrast, granular in-match performance statistics—including serve metrics, aces, return efficiency, and match duration—became systematically available only from 1991 onward with the ATP’s adoption of comprehensive statistical tracking^24^. For these metrics, we employ 5-year windows from 1991–2025 (*∼*98,000 matches). Decade-based windows over this span would yield only three full eras and just two consecutive era pairs, which is insufficient for sequential model evaluation. Five-year windows offer a natural compromise, as two such windows compose a decade, preserving alignment with our broader era-based framework, while providing sufficient data points for trend analysis. Shorter windows (e.g., 3 years) risk noisy distribution estimates due to reduced sample sizes. Additionally, this windowing strategy produces seven eras and six consecutive pairs with approximately 12,000–17,000 matches each, providing sufficient temporal depth for both distributional drift tracking and cross-era model evaluation^25^. Network analysis adopts the same five-year granularity over the full period, as centrality metrics such as hub scores and PageRank benefit from finer temporal resolution to capture structural shifts in player hierarchy.

Additionally, we adopted discrete (non-overlapping) temporal windows rather than rolling windows so that each era constitutes a clearly defined, independent analytical unit, facilitating direct era-to-era comparisons of distributions. Rolling windows, while offering smoother temporal trajectories, introduce autocorrelation between adjacent periods that complicates statistical comparison of distributional differences^26^. The boundary windows—1968–1975 (8 years) and 2021–2025 (5 years)—deviate from the nominal spans due to the start of the Open Era and the data extraction cutoff, respectively. Both are retained to ensure complete temporal coverage; their non-standard durations are acknowledged as a limitation.

For each match, three categories of information were extracted:

- *Player characteristics:* age, height, handedness, nationality, and ranking.
- *Match metadata:* tournament name, level (e.g., Grand Slam, Masters 1000), round, draw size, surface, and date.
- *In-match statistics:* aces, double faults, serve/return points won, service games, break points, and match duration (available systematically from 1991 onward).

### Concept drift as an analytical framework

Our analytical framework combines competitive balance analysis with concept drift methodologies^27^. Competitive balance is assessed through network centrality metrics that capture structural shifts in player hierarchy. Concept drift analysis comprises two complementary components: (i) distributional shift quantification via Jensen–Shannon divergence and Wasserstein distance to measure changes in *P*(*X*) across eras, and (ii) concept drift assessment via conditional probability estimation and cross-era predictive model evaluation to assess changes in *P(Y ∣ X)*. All analyses are stratified by surface type to account for surface-based trends in performance and drift patterns.

#### Competitive balance analysis

To evaluate how competitive dynamics have evolved over time, we analyzed matches grouped into temporal windows spanning the full Open Era and computed outcome-based and network-level metrics. Outcome-based measures, computed at decade-level resolution, capture the balance and intensity of competition. A **straight-set win** is defined as a match in which the winner did not lose a set, while a **heavy defeat** refers to a straight-set match where the loser won at most three games in total—at this margin, the loser averaged fewer than two games per set, indicating near-complete dominance. **Upsets** are matches in which a lower-ranked player defeated a higher-ranked opponent^28^, further categorized into **major upsets** (ranking differential *≥* 100) and **regular upsets** (all remaining upsets), representing two distinct competitive tiers. The 100-position threshold reflects an institutionally meaningful boundary in professional tennis: the top *∼*100 players receive direct entry into Grand Slam and major tour-level events^29^, and empirical evidence shows that win probability for the higher-ranked player plateaus beyond a ranking gap of approximately 80 positions^30^, making upsets beyond this range genuinely exceptional. **Higher-ranked wins** represent matches won by the player with the higher ranking, reflecting expected outcomes. Matches extending beyond straight sets are classified as **non–straight-set** matches, indicating more competitive and closely contested encounters.

To quantify dominance patterns at finer temporal resolution, each approximately five-year era was modeled as a directed network where nodes represent players and weighted edges (winner *→* loser) reflect victory counts. From these networks, we computed centrality-based dominance metrics following established methodologies in tennis analytics^23,31^. **Hub scores** (HITS algorithm) quantify success against strong opponents. Let *A* be the adjacency matrix weighted by match victories. Hub scores $\textbf{h}$ and authority scores $\textbf{a}$ satisfy $\textbf{h} = A \textbf{a}$, $\textbf{a} = A^\top \textbf{h}$, with $L_1$-norm normalization $|\textbf{h}|_1 = |\textbf{a}|_1 = 1$, ensuring that both hub and authority vectors sum to unity. **Out-degree centrality** measures winning reach across unique opponents: $c_{\text {out}}(v) = \frac{\deg _{\text {out}}(v)}{n-1}$, where $\deg _{\text {out}}(v)$ is the weighted out-degree of player *v* and *n* is the total number of players. **PageRank** captures prestige weighted by opponent strength: $\textbf{p} = (1 - d)\frac{1}{n}\textbf{1} + dA^\top \textbf{p}$, with damping factor *d=0.85* and normalization $\sum _i p_i = 1$. From these centrality measures, we derived dominance concentration metrics to distinguish eras of concentrated dominance from those with greater competitive parity: (i) *Top-10 hub share*–the proportion of total hub mass held by the ten highest-scoring players; (ii) *Dominance ratio*–the ratio of maximum hub score to mean hub score; (iii) *Mean out-degree*–the overall breadth of wins across the player field; and (iv) *Maximum PageRank*–the highest prestige level achieved within an era. Together, these metrics translate individual match outcomes into structural measures of competitive inequality.

#### Distributional shifts in tennis match statistics

To detect shifts in match feature distributions over time, we employed Jensen-Shannon (JS) divergence as our primary metric^32^. JS divergence offers several advantages for longitudinal sports analytics: it is symmetric (treating both distributions equally), bounded in the range [0,1] (facilitating interpretability across different features), and remains well-defined even when distributions have non-overlapping support. This was also complemented by the Wasserstein distance, selected for its ability to quantify geometric and transport-based differences in continuous distributions, providing sensitivity to location and shape variations that JS might overlook^33^. Probability density functions for each feature were estimated using kernel density estimation, applied to data aggregated into 5-year eras from 1991 to 2025. This dual-metric approach enabled robust identification of distributional changes in features such as match duration, ace rates, serve effectiveness, and player characteristics across different court surfaces.

#### Machine learning analysis of tennis performance evolution

In tennis analytics, best practices for constructing player features focus on feature differences to model tennis matches^34^. Predictive models typically use comparisons of player attributes and performance metrics derived from historical data, such as rank or average ace rate differences, to capture relative strengths between opponents. To investigate how the relationship between match features and outcomes has evolved–changes in *p(y=1 ∣ X)* where *y=1* indicates player 1 winning–we employed two complementary methods^24^. First, we estimated conditional win probabilities for key features (rank differences, age differences) using a non-parametric approach. Match data within each era were grouped into quantile-based bins and win proportions were calculated within each bin. These proportions were smoothed using Locally Weighted Scatterplot Smoothing (LOWESS)^35^, with logistic sigmoid transformation ensuring probabilities remained within [0,1]. Bootstrap resampling generated confidence intervals to quantify uncertainty in the estimated relationships.

Second, we evaluated historical predictive model degradation across eras using XGBoost classifiers, selected for their strong performance in the literature and their ability to capture non-linear relationships in sports data^36^. Models were trained to predict match winners based on differences in player profiles, including rank, age, serve and return efficiency metrics (first serve points won, return points won), aces, and double faults. To quantify concept drift, we adopted a sequential temporal validation framework^26^: for each consecutive pair of eras, we trained an XGBoost classifier on all matches from the earlier era (e.g., 1991–1995) and evaluated its performance both on the training era (using a validation split) and on the subsequent era (e.g., 1996–2000). This process was repeated across all six consecutive era pairs from 1991–2025. Model performance was assessed using accuracy and AUC metrics. Degradation was quantified as the difference between within-era performance (training era) and cross-era performance (subsequent test era), with negative values indicating performance drops. In this setup, the primary quantity of interest is not the within-era AUC but the magnitude of its drop when a model trained on one era is applied to a subsequent era, which directly measures how much the feature–outcome relationship has shifted—a classical and standard protocol for assessing concept drift^17,27^.

## Results

### Competitive balance: rising parity in modern tennis

**Fig. 1:**
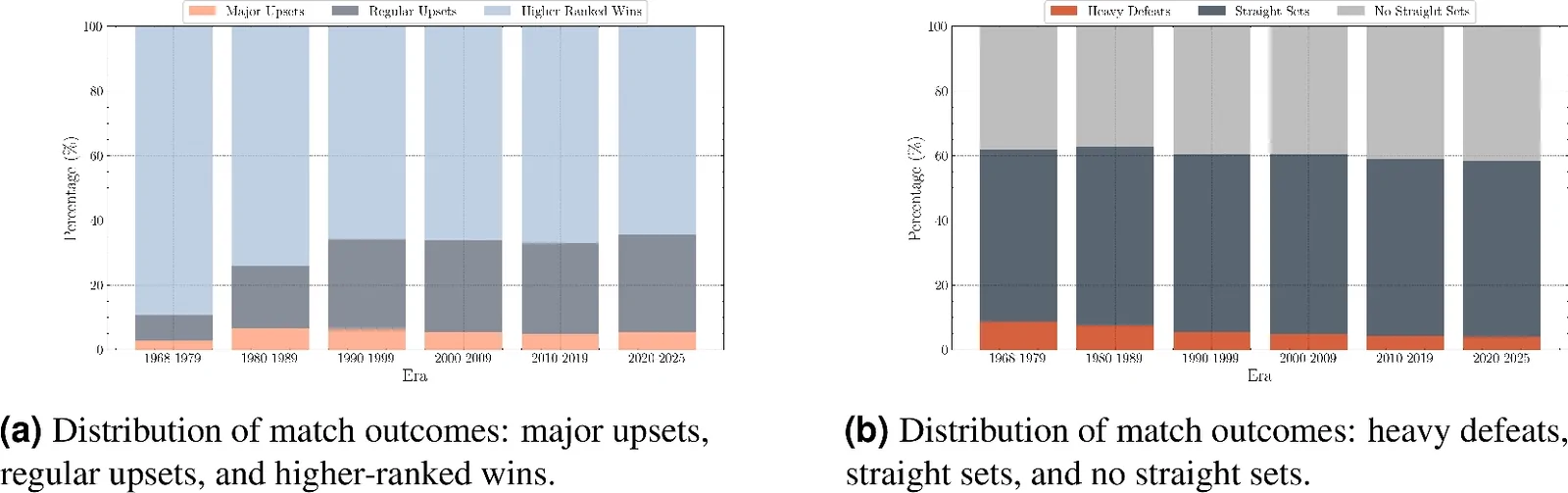
Competitive balance trends in men’s tennis since the Open Era.

Analysis of ATP Tour match outcomes (Fig. 1) reveals increasing competitive balance from 1968 to 2025. Upset proportions (lower-ranked player wins) rose from 10.7% in 1968–1979 to 35.5% in the most recent era, while major upsets (rank difference *≥*100) grew from 2.9% to 5.5%. Conversely, heavy defeats (straight-set matches where the loser won at most 3 games total) decreased from 8.6% to 3.9%, and straight-set proportions declined from 61.9% to 58.4%. The increase in upsets was not monotonic: upset rates plateaued during the 2000–2019 period before rising again in the most recent era.

**Fig. 2:**
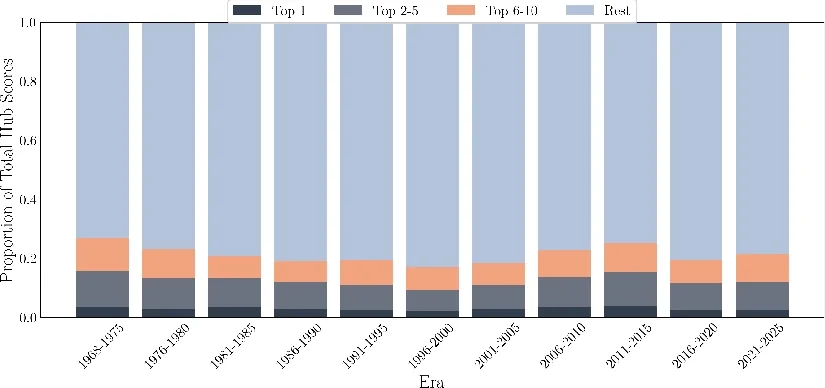
Proportion of hub scores by player tiers across eras.

**Fig. 3:**
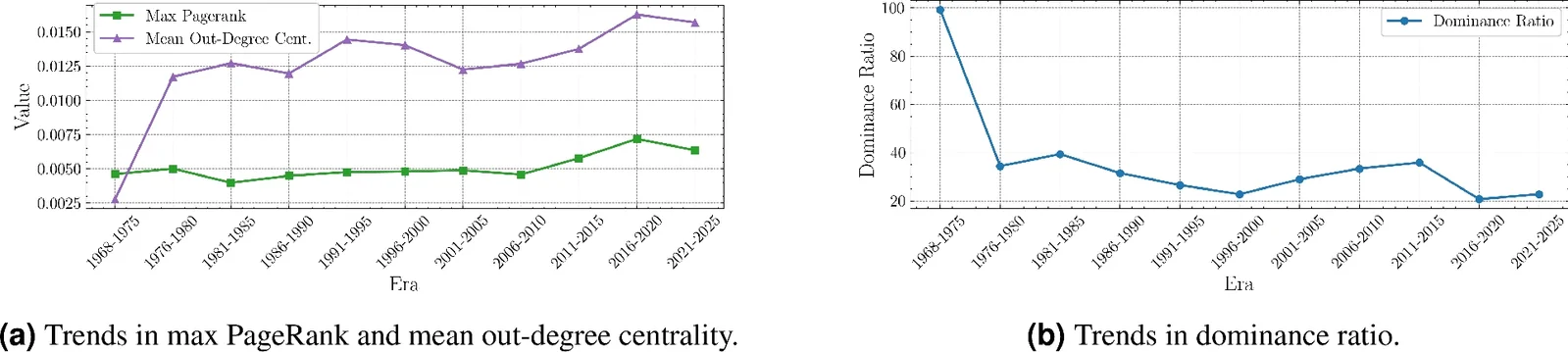
Trends in network-based metrics across eras.

Network-based concentration metrics show a similar pattern: top-10 hub concentration dropped from 27% to 22% of total hub mass, and the dominance ratio (max hub/mean hub) fell from *∼*99 (1968–1975) to *∼*23 (2021–2025). Figure 2 shows the proportion of hub scores by player tiers across eras, with hub concentration peaking during the 2011–2015 period. Figure 3 summarizes trends in max PageRank and mean out-degree centrality (Fig. 3a), and dominance ratio (Fig. 3b); max PageRank peaked in 2016–2020, while mean out-degree centrality rose steadily to its highest level in the most recent era. Table 1 lists the top-10 players per era by hub score, showing a transition from repeated names across consecutive eras (e.g., Lendl, McEnroe) to greater turnover in recent periods.

**Table 1:** Top 10 players by hub score per era. Hub scores are normalized (0–1 scale); higher values indicate greater dominance breadth (ability to beat diverse opponents).

	1968–1975		1976–1980		1981–1985		1986–1990		1991–1995		1996–2000	
	Name	Score	Name	Score	Name	Score	Name	Score	Name	Score	Name	Score
1	Rod Laver	0.0353	Jimmy Connors	0.0304	Ivan Lendl	0.0367	Ivan Lendl	0.0287	Pete Sampras	0.0264	Yevgeny Kafelnikov	0.0232
2	Arthur Ashe	0.0337	Björn Borg	0.0273	John McEnroe	0.0321	Stefan Edberg	0.0286	Michael Stich	0.0222	Pete Sampras	0.0200
3	John Newcombe	0.0310	Guillermo Vilas	0.0260	Jimmy Connors	0.0264	Boris Becker	0.0265	Jim Courier	0.0207	Marcelo Rios	0.0171
4	Tom Okker	0.0300	Brian Gottfried	0.0250	Mats Wilander	0.0218	Mats Wilander	0.0183	Stefan Edberg	0.0202	Tim Henman	0.0166
5	Stan Smith	0.0266	Eddie Dibbs	0.0240	Tomas Smid	0.0168	Brad Gilbert	0.0177	Michael Chang	0.0199	Alex Corretja	0.0165
6	Ken Rosewall	0.0260	Harold Solomon	0.0231	Guillermo Vilas	0.0165	Emilio Sanchez	0.0159	Boris Becker	0.0185	Thomas Enqvist	0.0164
7	Ilie Nastase	0.0256	John McEnroe	0.0207	Yannick Noah	0.0152	Andres Gomez	0.0146	Goran Ivanisevic	0.0183	Andre Agassi	0.0156
8	Marty Riessen	0.0233	Wojtek Fibak	0.0189	Andres Gomez	0.0147	Miloslav Mecir	0.0145	Sergi Bruguera	0.0175	Greg Rusedski	0.0154
9	Cliff Drysdale	0.0199	Vitas Gerulaitis	0.0188	Eliot Teltscher	0.0140	John McEnroe	0.0143	Thomas Muster	0.0164	Patrick Rafter	0.0153
10	Cliff Richey	0.0191	Raul Ramirez	0.0188	Johan Kriek	0.0132	Jakob Hlasek	0.0132	Andre Agassi	0.0162	Carlos Moya	0.0152

	2001–2005		2006–2010		2011–2015		2016–2020		2021–2025			
	Name	Score	Name	Score	Name	Score	Name	Score	Name	Score		
1	Roger Federer	0.0287	Roger Federer	0.0345	Novak Djokovic	0.0404	Novak Djokovic	0.0264	Jannik Sinner	0.0269		
2	Andy Roddick	0.0237	Rafael Nadal	0.0335	Roger Federer	0.0305	Rafael Nadal	0.0252	Carlos Alcaraz	0.0257		
3	Lleyton Hewitt	0.0216	Novak Djokovic	0.0272	Rafael Nadal	0.0291	Dominic Thiem	0.0227	Daniil Medvedev	0.0240		
4	Carlos Moya	0.0183	Andy Murray	0.0221	Andy Murray	0.0275	Alexander Zverev	0.0224	Alexander Zverev	0.0232		
5	Andre Agassi	0.0172	Nikolay Davydenko	0.0210	David Ferrer	0.0274	Roger Federer	0.0198	Novak Djokovic	0.0220		
6	Carlos Ferrero	0.0171	Andy Roddick	0.0205	Tomas Berdych	0.0251	David Goffin	0.0174	Stefanos Tsitsipas	0.0203		
7	Guillermo Coria	0.0157	David Ferrer	0.0200	Stan Wawrinka	0.0193	Roberto Bautista Agut	0.0165	Taylor Fritz	0.0197		
8	Marat Safin	0.0145	Robin Soderling	0.0183	Jo-Wilfried Tsonga	0.0192	Marin Cilic	0.0161	Andrey Rublev	0.0196		
9	Tim Henman	0.0145	Tomas Berdych	0.0162	Kei Nishikori	0.0179	Kei Nishikori	0.0152	Casper Ruud	0.0188		
10	Gaston Gaudio	0.0143	Fernando Verdasco	0.0160	Richard Gasquet	0.0162	Daniil Medvedev	0.0147	Alex de Minaur	0.0170		

### Transformative trends in tennis match performance

**Fig. 4:**
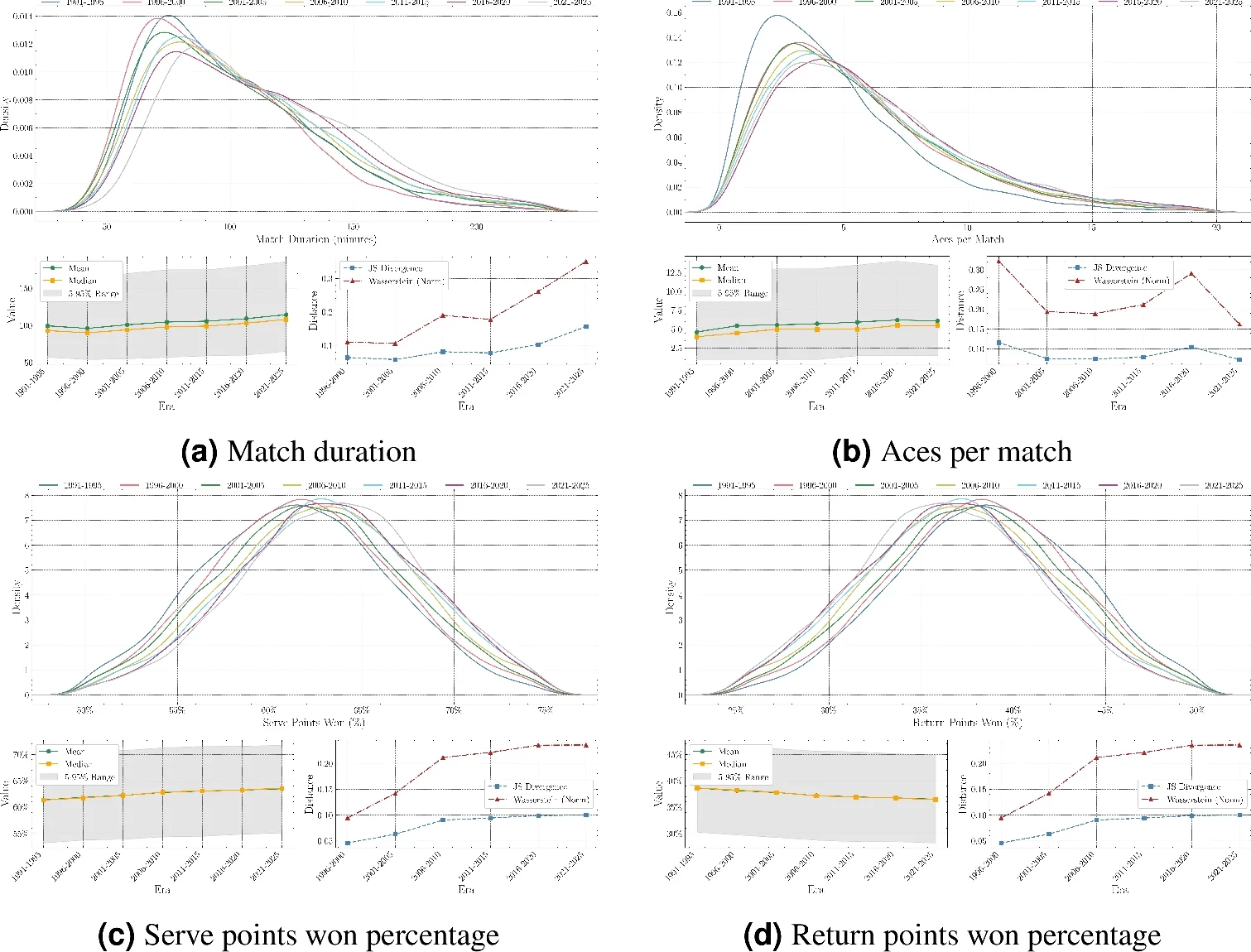
Distributional evolution and mean trends of key performance metrics across eras (1991–2025) for all surfaces.

Match duration exhibited one of the most pronounced upward trends across all performance metrics, accumulating a cumulative increase of +16.5 minutes between 1991–1995 and 2021–2025 (Fig. 4a). The increase was gradual across most eras but steepened between 1996–2000 and 2001–2005. Both JS divergence and normalized Wasserstein distance reached their highest values in the most recent era, indicating a shift in central tendency and a broadening of the duration distribution toward more varied match lengths. Serve-related metrics show a consistent upward trajectory. Aces per match increased by approximately +1.7 over the study period, and serve points won rose by +2.4 percentage points, with the largest distributional shifts in aces appearing during the earliest era transition (Fig. 4b and c). The rate of change in aces was steepest in the 1990s and flattened in later eras. Return points won declined by −2.4 percentage points, with its distributional drift instead intensifying in the more recent eras (Fig. 4d). Notably, the serve and return trajectories mirror each other almost exactly in magnitude, with serve gains and return losses accumulating at comparable rates across successive eras.

### Shifts in tennis winning factors

**Fig. 5:**
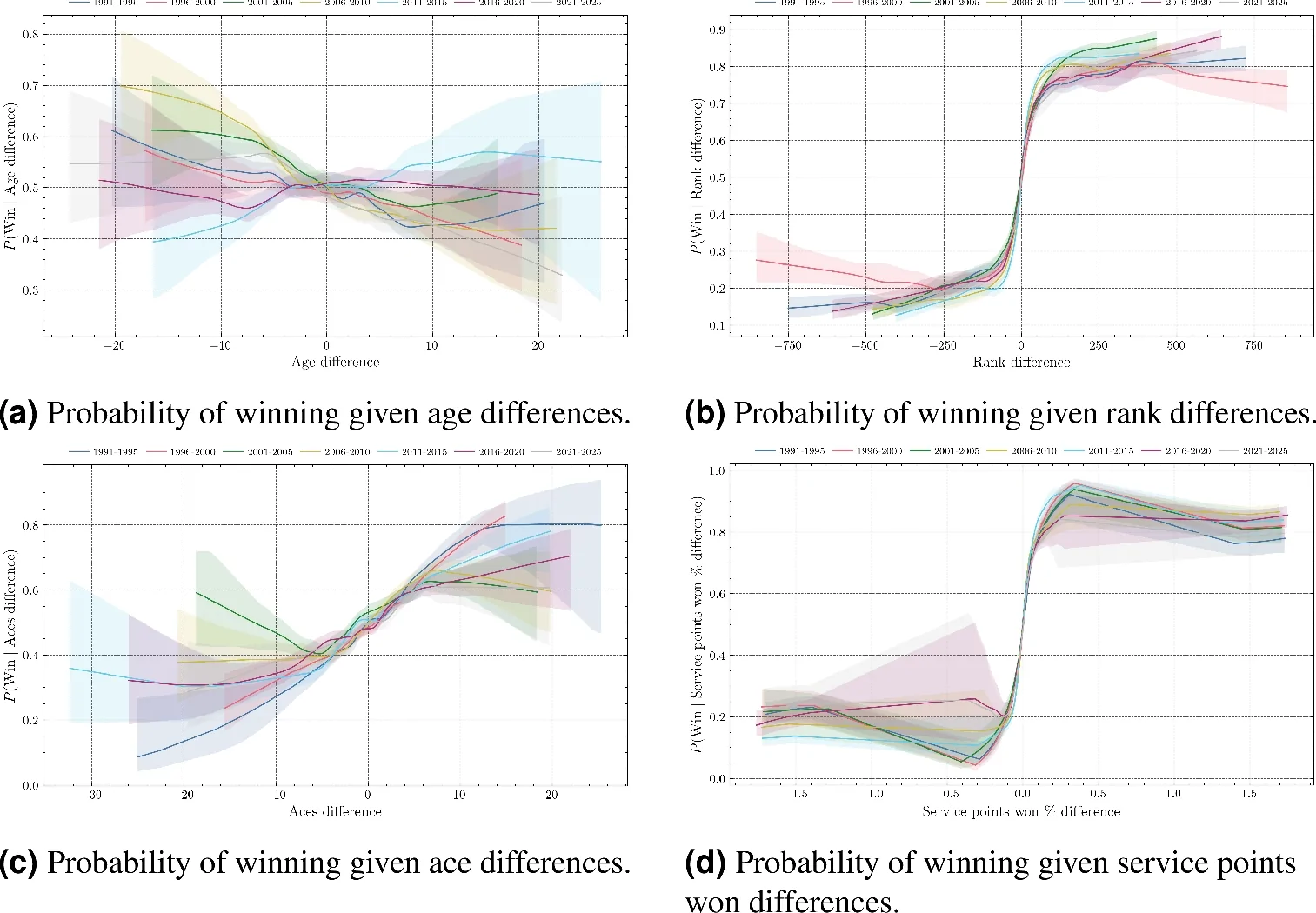
Conditional probability curves illustrating shifts in winning factors across eras (1991–2025). Each subplot shows the relationship between a match feature and win probability, smoothed using LOWESS.

Conditional probability curves (Fig. 5) reveal era-dependent shifts in how match features relate to outcomes. The win probability associated with an age advantage declined overall from the earliest to the most recent era, but exhibited a temporary reversal during 2011–2020 when older, top-ranked players maintained elevated win rates (Fig. 5a). Rank differences showed the opposite trajectory: their predictive power strengthened through the mid-2000s before partially reverting in recent eras (Fig. 5b). Ace differences displayed modest and periodic fluctuations, with a slight increase in predictive value during the early 2000s (Fig. 5c). In contrast, service points won differences remained the most stable feature-outcome relationship across all eras, with consistently high win probabilities for even small advantages (Fig. 5d).

**Fig. 6:**
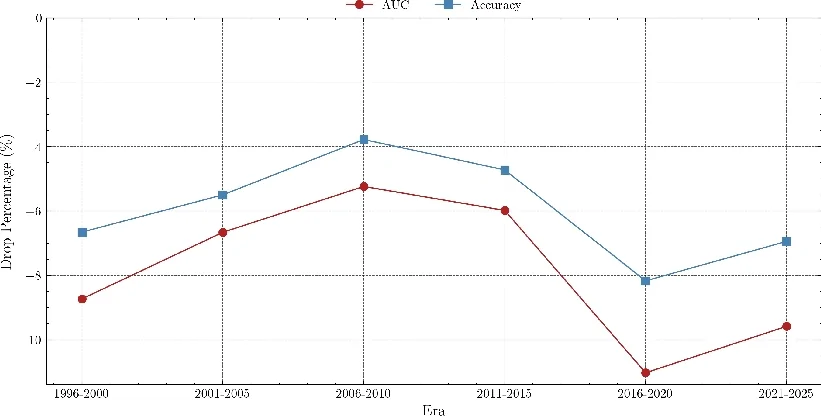
Model performance degradation (AUC and accuracy drops) across eras.

Cross-era model evaluation revealed consistent performance degradation across all six era transitions (Fig. 6). AUC drops ranged from roughly 6% to 11%, with the largest drop at the 2011–2015 to 2016–2020 boundary. Accuracy degradation followed a similar pattern. No era transition exhibited zero degradation, confirming that a model calibrated on any given five-year window systematically lost predictive accuracy when applied to the subsequent window.

### Surface-specific evolution: divergent trends across playing conditions

**Fig. 7:**
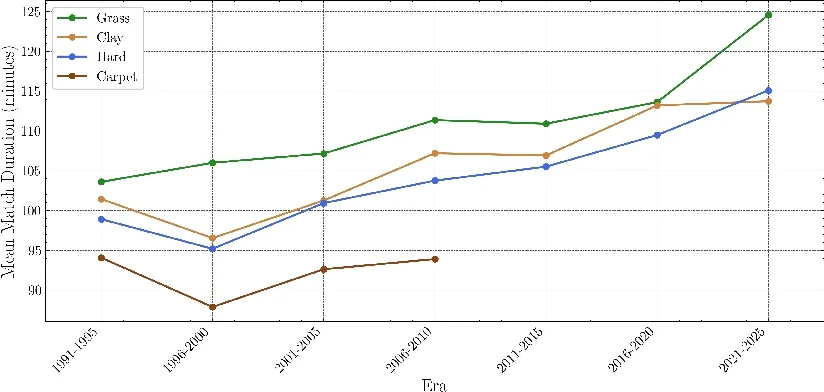
Mean match duration trends across surfaces and eras.

We stratified the analysis by court type (Grass, Clay, Hard, Carpet). Mean match duration (Fig. 7) increased across all active surfaces, but the magnitude varied substantially. Grass courts showed the largest cumulative rise (+21.0 minutes), followed by Hard (+16.2 minutes) and Clay (+12.3 minutes), while Carpet remained largely stable before its phase-out post-2010. By the most recent era, Grass and Hard court durations converged to similar levels despite their historically distinct playing speeds, whereas Clay maintained a shorter average duration throughout.

**Fig. 8:**
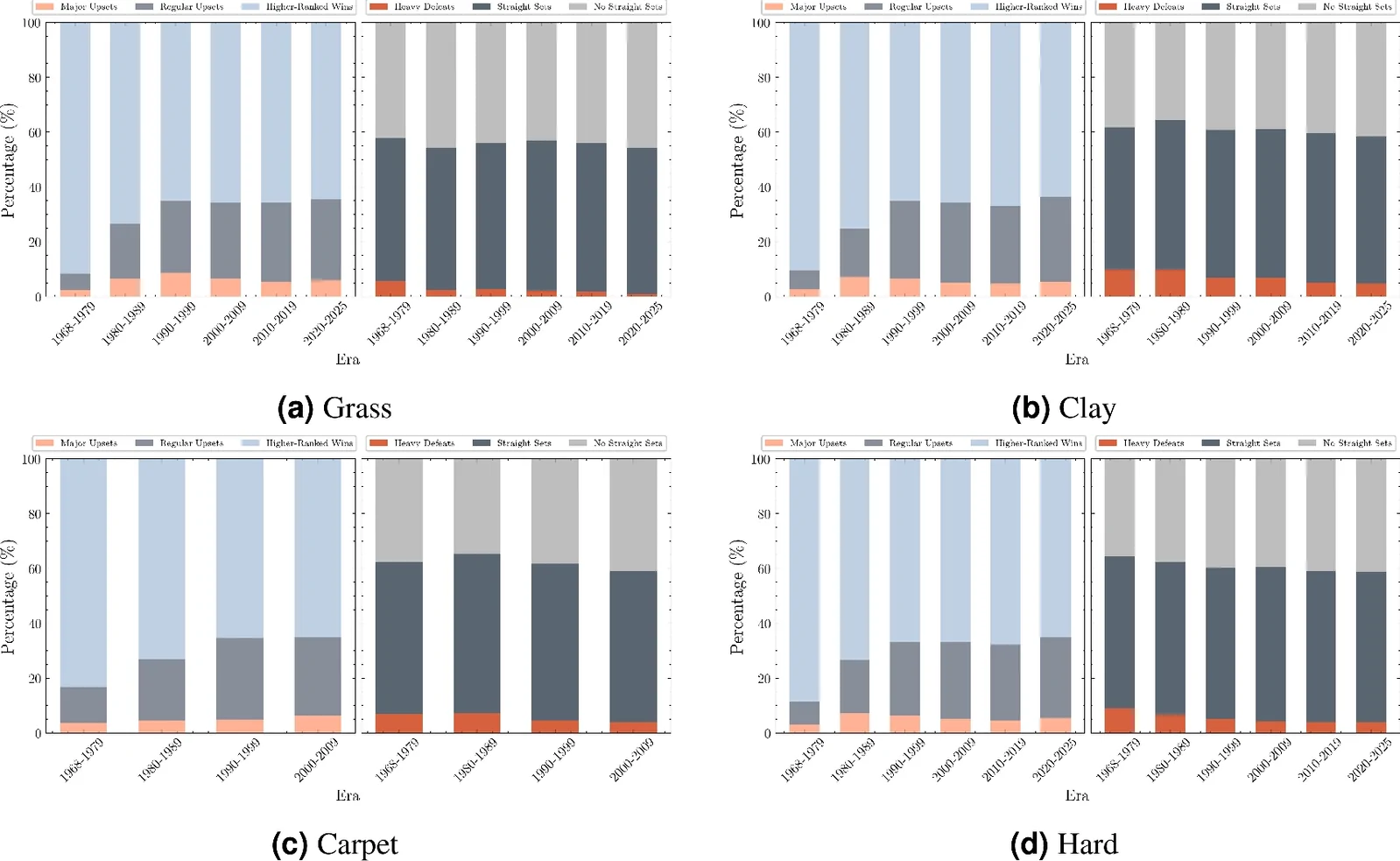
Competitive balance trends (upsets, heavy defeats, straight sets, major upsets) across surfaces (1968–2025).

Surface-specific competitive balance metrics (Fig. 8) show upsets increasing on every surface, with all four court types converging toward approximately 35% by the most recent era despite starting from different baselines. Grass exhibited the largest absolute increase in upset frequency and the highest incidence of major upsets, which peaked during the 1990s. Straight-set victories declined uniformly across all surfaces. The inter-surface spread in upset rates narrowed over time, with surfaces that originally had the lowest upset rates (Grass and Clay) catching up to Hard and Carpet levels.

**Fig. 9:**
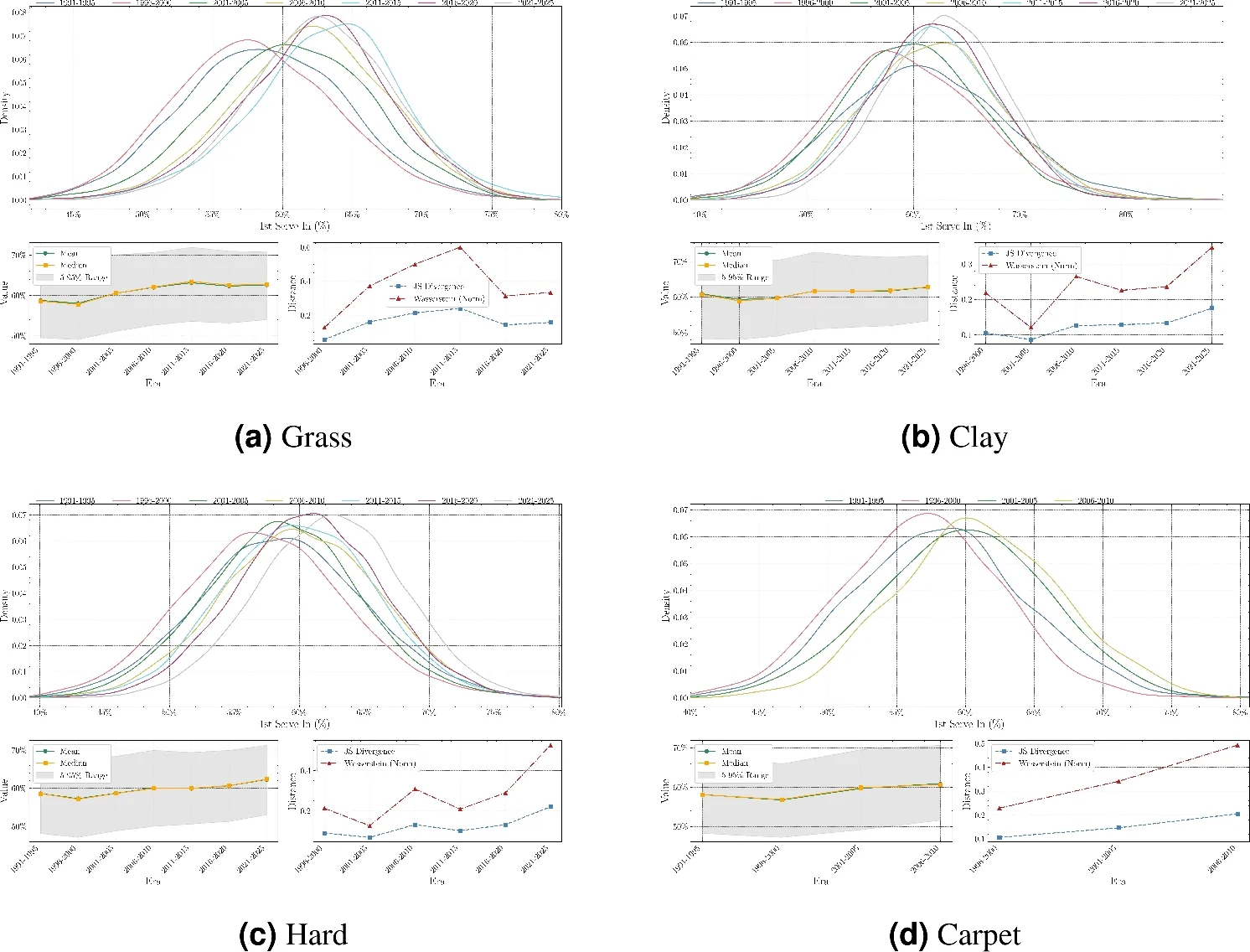
Distributional evolution and mean trends of 1st serve in percentage across surfaces (1991–2025).

**Fig. 10:**
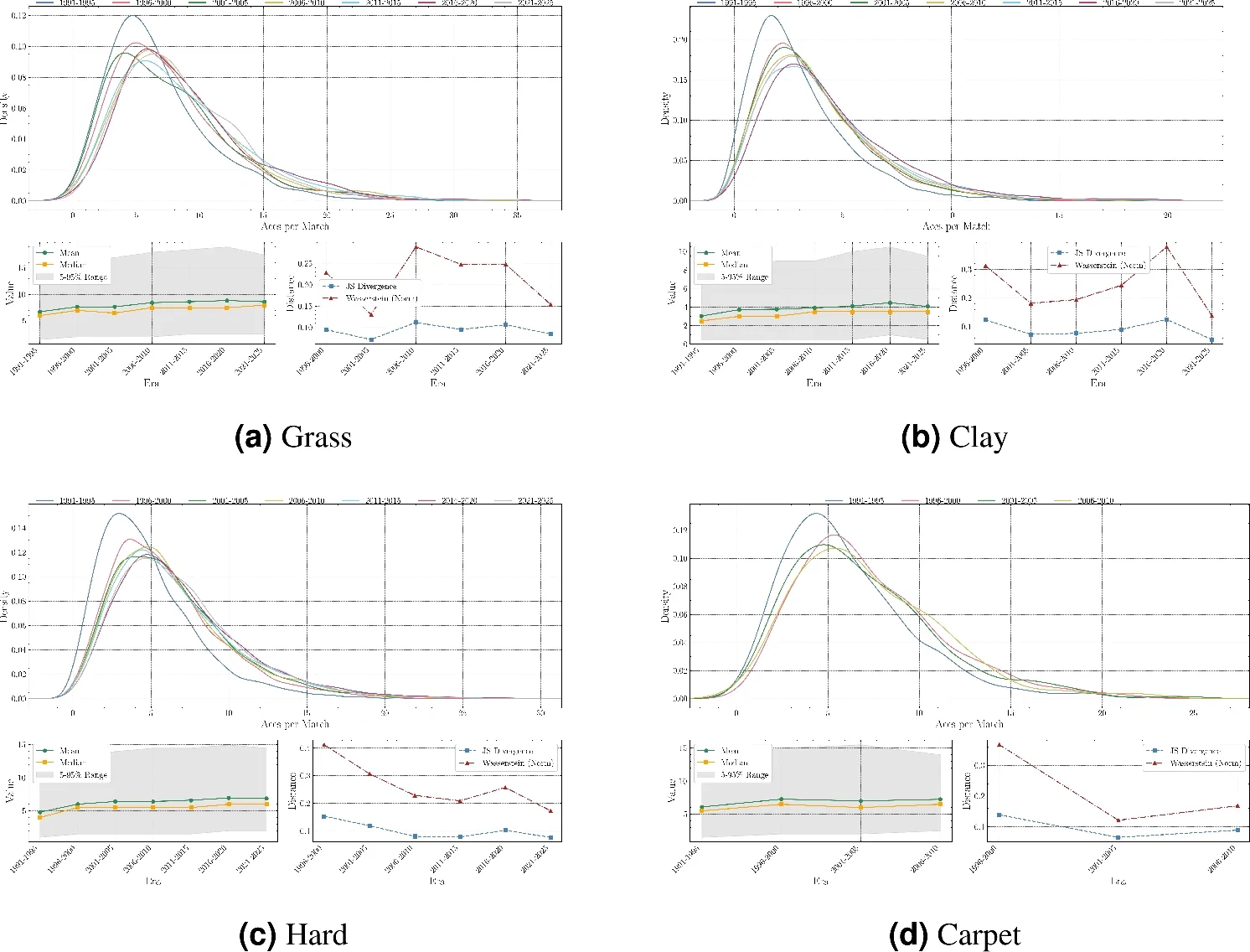
Distributional evolution and mean trends of aces per match across surfaces (1991–2025).

**Fig. 11:**
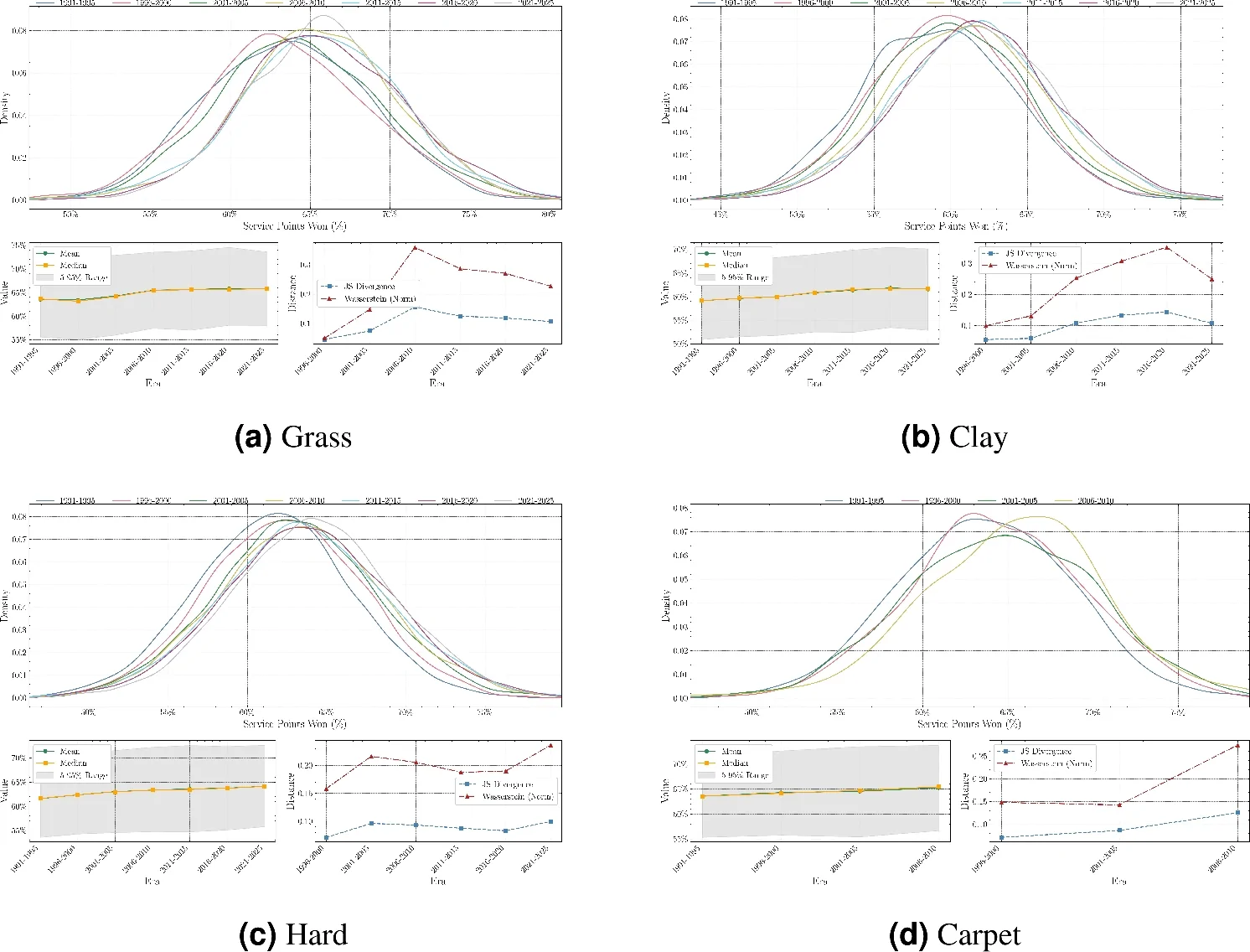
Distributional evolution and mean trends of service points won percentage across surfaces (1991–2025).

First-serve-in percentage (Fig. 9) increased on all surfaces, with the largest gains on Grass and Hard (approximately +3.7–3.9 percentage points each) and smaller increases on Clay and Carpet. Aces per match (Fig. 10) grew most on Hard (+2.1) and Grass (+1.9), roughly double the increase on Clay and Carpet. Service points won percentage (Fig. 11) rose by approximately 2–2.5 percentage points across all active surfaces. An asymmetry is visible across these metrics: the inter-surface gap in ace rates widened over time, with fast surfaces pulling further ahead, whereas the gap in overall serve-point efficiency narrowed, indicating that serve-point outcomes became more similar across surfaces even as ace counts diverged.

## Discussion and conclusions

This study provides a comprehensive quantitative framework for understanding the evolution of men’s professional tennis from 1968 to 2025, revealing fundamental transformations in competitive balance, performance metrics, and match dynamics. Our findings indicate rising parity across the sport, evidenced by increased upsets (from 10.7% to 35.5%) and reduced heavy defeats (from 8.6% to 3.9%). The notably low upset rate in the earliest era (10.7% in 1968–1979) should be interpreted with caution, as ATP rankings were not introduced until 1973; prior to that, ranking data were not used formally, which may understate the true frequency of upsets in this period. These trends are accompanied by longer match durations (+16.5 minutes cumulatively) and shifts toward greater serve efficiency (aces +1.7, service points won +2.4%). The symmetric decline in return points won (−2.4%) mirrors these serve gains, pointing to a progressive offensive emphasis where serve effectiveness increasingly outpaces return capability. Surface-specific trends show homogenization effects, with grass and hard courts converging toward similar match durations and exhibiting the strongest distributional drifts in duration and aces (JS exceeding 0.15 for grass duration and hard-court aces), while clay maintains its characteristic endurance focus. Concept drift analysis confirms these changes through distributional shifts in serve/return metrics and model degradation (AUC drops up to 11%). No era transition showed zero performance loss, indicating that feature-outcome relationships vary across eras. For instance, rank predictability strengthened during periods of concentrated dominance (win probability rise from 0.547 to 0.582 for +10-rank advantages from 1991–1995 to 2006–2010), while age-based advantages declined overall despite temporary spikes during the Big Three era. Network metrics further show declining individual dominance (dominance ratio from  99 to  23) and broader field competitiveness (mean out-degree to  0.016), suggesting a sport adapting to professionalization with implications for both competitive parity and physical endurance demands^37^.

The period from the early 2000s to late 2010s, dominated by R.Federer, R.Nadal, and N.Djokovic–who collectively won 66 Grand Slam titles between 2003 and 2023–represents a notable deviation from the broader trend toward parity. Our network analysis captures this influence through elevated hub concentration and prestige metrics during these years. The top-10 hub share rises to 25% in 2011–2015 (Fig. 2), its highest level since the early Open Era, reflecting a top-heavy structure where the Big Three accumulated disproportionate dominance. Max PageRank reaches its highest values in 2016–2020 (0.0072), indicating that victories increasingly came against high-quality opponents as they frequently faced each other in late tournament stages. This concentration correlates with our outcome-based metrics, where upsets dipped slightly during their peak years, and the dominance ratio spikes to  36 in 2011–2015 (Fig. 3). These patterns extend to match performance trends, with extended durations (+16.5 minutes cumulatively since 1991, Fig. 4a) and heightened serve efficiency (service points won reaching 63.6%, Fig. 4c) reflecting the endurance and tactical precision demanded by their prolonged rivalries. However, recent eras (2021–2025) show declining concentration metrics, with rising mean out-degree centrality ( 0.016) and a broader competitive elite including emerging stars like Alcaraz, Sinner, and Zverev, signaling a transition to greater field-wide competitiveness as the Big Three’s influence wanes.

These performance shifts unfolded alongside notable changes in racket engineering and surface preparation^38^. Racket design has evolved dramatically since the 1970s, transitioning from wooden frames to fiber-polymer composites offering greater stiffness, larger head sizes, and reduced mass while maintaining or enhancing power^1^. Analysis of 525 rackets from 1874 to 2017 reveals that pre-1970 wooden rackets featured head areas below 0.05 m², masses over 350 g, and natural frequencies below 120 Hz, constraining power and maneuverability. In contrast, post-1980 composite rackets exhibited larger heads (r = 0.897 with principal component analysis, p < 0.001), lower masses, and higher natural frequencies (r = 0.813, p < 0.001), enabling faster serves and more aggressive baseline play^1^. Our analysis shows that the rise in aces and serve effectiveness spans the same interval, particularly on fast surfaces where JS divergences for individual era transitions reached 0.10–0.15 (Figs. 10a,c and 11a,c). While our study does not directly measure equipment effects, the temporal correspondence between composite racket adoption and the +2.1 ace increase on hard courts, alongside the cumulative −2.4% decline in return points won (Fig. 4d), is consistent with the biomechanical advantages documented in the literature^1^.

A parallel development is court speed homogenization. The ITF’s 2002 introduction of varied ball types was designed to reduce rally duration variances across surfaces^39^, and our surface-specific results are consistent with this objective. For instance, the use of larger balls on fast courts and harder balls on slow ones has diminished surface-specific disparities, as evidenced by reduced differences in rally durations post-2002^40^. This shift is clearly reflected in our surface-specific analysis, grass now displays the strongest rise in match duration (+21.0 minutes cumulatively, Fig. 7) and the largest distributional displacement (JS = 0.162 in 2021–2025), marking its transition from serve-and-volley dominance toward baseline-oriented exchanges^7^. Recent meta-analyses substantiate that these adjustments prolong rally lengths and amplify physical demands, with elite (international) males experiencing longer rallies on clay (approximately 7.1 s [6.2, 8.1]) compared to grass (4.3 s [3.1, 5.9]) and covering greater distances more efficiently than sub-elite (national/junior) players, who record higher total distances due to measurement variations^39^. Systematic reviews on serve biophysics further outline demands, including average distances of around 2.3 km in best-of-five set matches for elite males on hard courts, coupled with peak accelerations of 3–4 m/s² during serves, which may heighten by contemporary racket technology and surface variations^41^. These physical demands align with the broader competitive balance trends observed in our analysis: reduced heavy defeats and increased upsets, as longer and more physically demanding matches may favour a wider range of competitive outcomes.

From a dynamical systems perspective, these technological and surface changes have reshaped player interactions as coupled oscillators. Tennis displacements can be modeled as periodic oscillations about baseline positions, with relative phase capturing synchronization between players^37^. Our observed drifts, such as increased match variability on homogenized surfaces (Wasserstein norm = 0.39 on grass in 2021–2025), suggest that slower courts and advanced rackets prolong these oscillatory patterns, leading to more frequent phase transitions and extended durations. A similar interplay between power and endurance emerges in prior work on visual coupling and synchronization^37^, where modern rackets enhance force generation while slower courts prolong exchanges. This dual influence echoes in our conditional probability trends: an additional +2 ace advantage increases win likelihood by 0.021 between 1996–2000 and 2001–2005 (Fig. 5c). Concept drift analysis further reveals that feature-outcome relationships are era-dependent, rendering historical predictive models less reliable and necessitating adaptive training and tactical approaches for coaches, while surface-specific trends amid homogenization reveal tennis as a complex adaptive system in which technology, strategy, and competition co-evolve.

This study establishes the first comprehensive quantitative baseline for tennis evolution through integrated concept drift analysis and network analysis. By quantitatively revealing that professional tennis has transitioned toward greater competitive parity, endurance-based performance, and surface homogenization, punctuated by temporary concentration during the Big Three era, we provide evidence-based insights for multiple stakeholders. For tournament organizers, the documented surface homogenization—particularly the +21.0-minute increase in grass-court match duration—suggests that scheduling and rest-day allocation may need revisiting as matches grow longer across all surfaces. The convergence in playing styles across surfaces also raises questions about whether further court-speed differentiation could enhance spectator variety. For coaches and performance analysts, the progressive model degradation (AUC drops up to 11%) demonstrates that training strategies calibrated on historical data lose predictive validity over relatively short periods; regular recalibration to current competitive conditions is essential. For example, the declining return-point efficiency (−2.4%) implies that return-focused training may need greater emphasis to counteract the growing serve advantage. For sports scientists, the +16.5-minute increase in mean match duration, combined with rising serve loads (aces +1.7 per match), points to evolving injury risk profiles; longitudinal studies linking these performance trends to injury incidence data could inform evidence-based workload management and recovery protocols.

### Limitations

Several limitations for this study should be acknowledged. First, ATP rankings were not introduced until 1973, so competitive balance metrics for the 1968–1979 era rely on incomplete ranking data. However, this affects only the earliest era; all post-1973 analyses use official rankings and the observed trends remain consistent. Second, the boundary eras (1968–1979 and 2021–2025) deviate from the nominal decade or five-year spans, though they are retained to ensure complete temporal coverage and sensitivity analyses confirm that excluding them does not alter trend directions. Third, the thresholds defining heavy defeats (*≤*3 games won) and major upsets (*≥*100 ranking-position differential) are grounded in competitive tiers and match structure but remain discretionary; while alternative thresholds would shift reported magnitudes, the underlying trend directions are robust across reasonable variations. Fourth, while we discuss temporal correspondences between our findings and external factors such as racket technology and ITF rule changes, these variables were not directly modeled; incorporating equipment specifications, court-speed measurements, and rule amendments as covariates would enable causal analysis and represents a natural extension of this work. Fifth, fixed-width temporal windows capture era-level trends but cannot isolate single-year transitions. The distributional metrics computed here—JS divergence and Wasserstein distance—provide a quantitative foundation that could be combined with finer-grained or event-driven segmentation to identify historically significant transition points in the sport’s evolution, opening promising avenues for sports historians and analysts seeking to pinpoint when and why the game changed.

## Data Availability

The match data analysed in this study are publicly available from the Tennis Abstract repository (https://github.com/JeffSackmann/tennis_atp) under a Creative Commons Attribution–NonCommercial–ShareAlike 4.0 International license. Derived data supporting the findings are available from the corresponding author upon reasonable request.

## Code availability

The custom code used to generate the results of this study is openly available on Zenodo (https://doi.org/10.5281/zenodo.20445481) under a Creative Commons Attribution–NonCommercial–ShareAlike 4.0 International license.
